# Supplementary material for: Multicenter evaluation of abbreviated MRI and ultrasound for detecting early-stage hepatocellular carcinoma
Source: JHEP Rep. 2025 Feb 12;7(5):101357. doi: 10.1016/j.jhepr.2025.101357 (PMC12048809; doi:10.1016/j.jhepr.2025.101357)
Supplement: Multimedia component 1 [file mmc1.pdf]

Multicenter evaluation of abbreviated MRI and ultrasound for detecting early-stage hepatocellular carcinoma

Karim Seif El Dahan, Takeshi Yokoo, Darine Daher, Matthew S. Davenport, David T. Fetzer, Mishal Mendiratta-Lala, Nicole E. Rich, Edward Yang, Neehar D. Parikh, Amit G. Singal

Table of contents

Fig. S1……………………………………………………………………………………………2

Figure S1: Patient Eligibility Flow Diagram

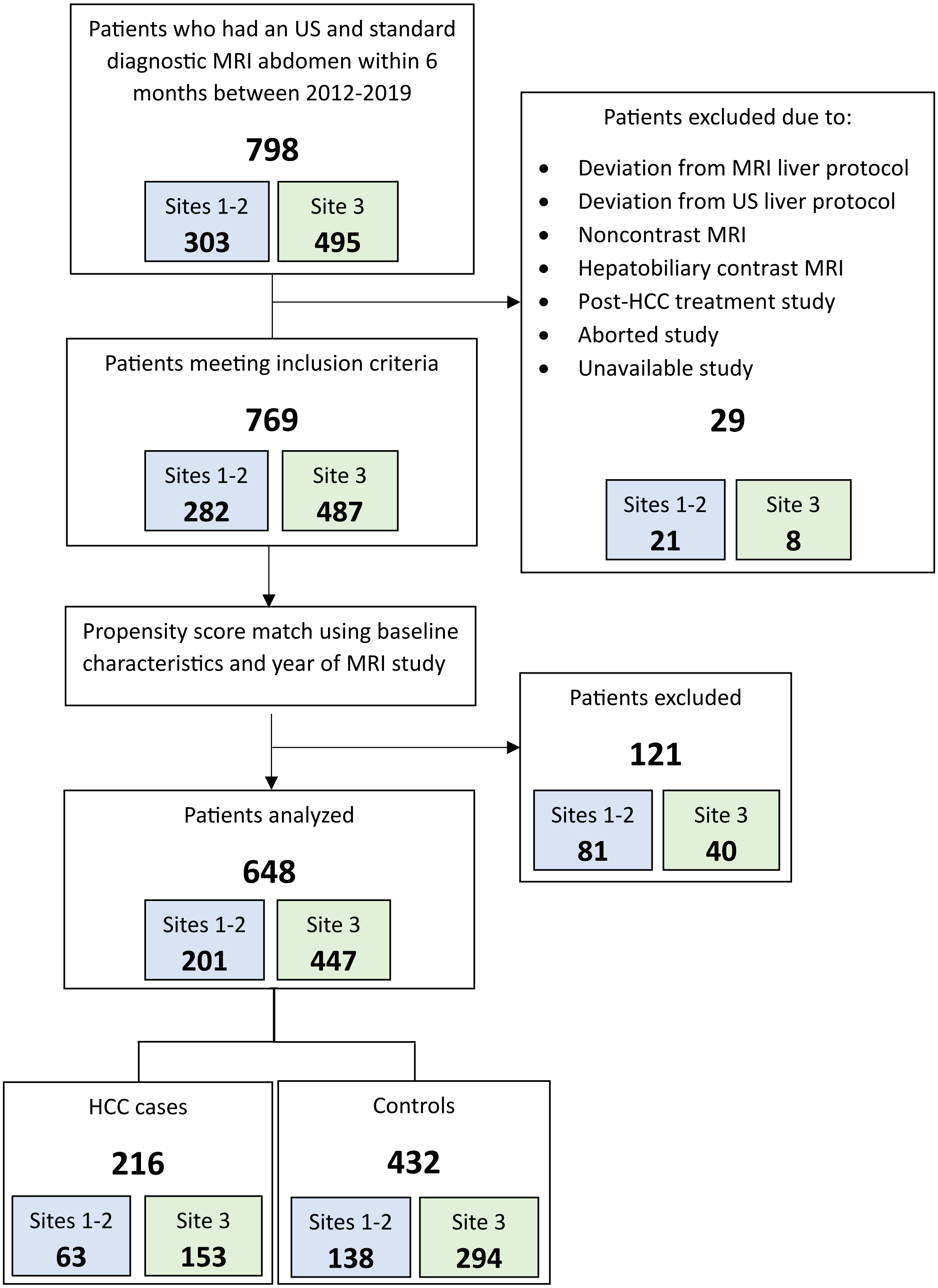

HCC = hepatocellular carcinoma.
