# Supplementary material for: Multicenter evaluation of abbreviated MRI and ultrasound for detecting early-stage hepatocellular carcinoma
Source: JHEP Rep. 2025 Feb 12;7(5):101357. doi: 10.1016/j.jhepr.2025.101357 (PMC12048809; doi:10.1016/j.jhepr.2025.101357)
Supplement: Multimedia component 2 [file mmc2.docx]

**JHEP Reports**

**CTAT methods**

Tables for a “Complete, Transparent, Accurate and Timely account” (CTAT) are now mandatory for all revised submissions. The aim is to enhance the reproducibility of methods.

- Only include the parts relevant to your study
- Refer to the CTAT in the main text as ‘Supplementary CTAT Table’
- Do not add subheadings
- Add as many rows as needed to include all information
- Only include one item per row

**If the CTAT form is not relevant to your study, please outline the reasons why:**

|  |
| --- |

- 1. **Antibodies**

| **Name** | **Citation** | **Supplier** | **Cat no.** | **Clone no.** |
| --- | --- | --- | --- | --- |
| N/A |  |  |  |  |

- 1. **Cell lines**

| **Name** | **Citation** | **Supplier** | **Cat no.** | **Passage no.** | **Authentication test method** |
| --- | --- | --- | --- | --- | --- |
| N/A |  |  |  |  |  |

- 1. **Organisms**

| **Name** | **Citation** | **Supplier** | **Strain** | **Sex** | **Age** | **Overall n number** |
| --- | --- | --- | --- | --- | --- | --- |
| N/A |  |  |  |  |  |  |

- 1. **Sequence based reagents**

| **Name** | **Sequence** | **Supplier** |
| --- | --- | --- |
| N/A |  |  |

- 1. **Biological samples**

| **Description** | **Source** | **Identifier** |
| --- | --- | --- |
| N/A |  |  |

- 1. **Deposited data**

| **Name of repository** | **Identifier** | **Link** |
| --- | --- | --- |
| N/A |  |  |

- 1. **Software**

| **Software name** | **Manufacturer** | **Version** |
| --- | --- | --- |
| R statistical software | R Core Team | 4.2.1. |
| REDCap | Vanderbilt University | 13.1.28 |

- 1. **Other (*e.g*. drugs, proteins, vectors etc.)**

| N/A |  |  |
| --- | --- | --- |
|  |  |  |

- 1. **Please provide the details of the corresponding methods author for the manuscript:**

| Amit G. Singal, M.D., M.S.  Division of Digestive and Liver Diseases  University of Texas Southwestern  5959 Harry Hines Blvd, POB 1, Suite 420  Dallas TX 75390-8887 USA  Tel: 214-645-6029  Fax: 214-645-6294  Email: [amit.singal@utsouthwestern.edu](mailto:amit.singal@utsouthwestern.edu) |
| --- |

**2.0 Please confirm for randomised controlled trials all versions of the clinical protocol are included in the submission. These will be published online as supplementary information.**

| N/A |
| --- |
