# Supplementary material for: Multicenter evaluation of abbreviated MRI and ultrasound for detecting early-stage hepatocellular carcinoma
Source: JHEP Rep. 2025 Feb 12;7(5):101357. doi: 10.1016/j.jhepr.2025.101357 (PMC12048809; doi:10.1016/j.jhepr.2025.101357)
Supplement: Multimedia component 4 [file mmc4.pdf]

JHEP|Reports

Multicenter evaluation of abbreviated MRI and ultrasound for detecting early-stage hepatocellular carcinoma

Karim Seif El Dahan[1,†], **Takeshi Yokoo**[2,†], Darine Daher[1], Matthew S. Davenport[3,4], David T. Fetzer[2], Mishal Mendiratta-Lala[3], Nicole E. Rich[1], Edward Yang[5], Neehar D. Parikh[6,‡], Amit G. Singal[1,*,‡]

JHEP Reports **2025**. vol. 7 | 1–10

Check for updates

Background & aims: Abbreviated MRI (AMRI) has been proposed as an alternative to ultrasound for hepatocellular carcinoma (HCC) surveillance; however, comparative data for AMRI and ultrasound are needed. Thus, we evaluated the sensitivity and specificity of dynamic contrast-enhanced (DCE)-AMRI and ultrasound for early-stage HCC detection in patients with cirrhosis.

Methods: We conducted a multicenter retrospective case–control study among patients with cirrhosis (cases with early-stage HCC as per Milan Criteria; controls without HCC) who underwent an ultrasound and a DCE-MRI within a 6-month period between 2012 and 2019. HCC diagnosis was confirmed by imaging alone in 85% and by histopathology in 15% of patients. Dynamic AMRI examinations were simulated from the full MRI by selecting relevant sequences. Independent, blinded interpretations of ultrasounds and AMRI results were performed using Liver Imaging Reporting and Data System algorithms. Ultrasounds were considered positive if US-3 observations were detected. AMRI was considered positive if LR-4, LR-5, or LR-M were detected. Per-patient sensitivity and specificity for early-stage HCC detection were estimated, and cross-modality differences were tested.

Results: We included 216 cases and 432 controls. Patient-level sensitivity and specificity of AMRI were significantly higher compared with ultrasound: 80.1% (95% CI 76.1–83.6) *vs.* 71.1% (95% CI 66.6–75.2), p <0.001, and 91.9% (95% CI 89.9–93.5) *vs.* 72.3% (95% CI 69.3–75.2), p <0.001, respectively. AMRI sensitivity was significantly higher compared with ultrasound among patients with Child-Pugh B cirrhosis (80.8% *vs.* 57.4%, p <0.001) but not among those with Child-Pugh A (84.7% *vs.* 78.6%, p = 0.07) or Child-Pugh C cirrhosis (52.6% *vs.* 68.4%, p = 0.18).

Conclusions: Dynamic AMRI may be more sensitive and specific for early-stage HCC detection in patients with cirrhosis compared with ultrasound, although its relative benefit might be smaller in patients with Child-Pugh A cirrhosis. Larger direct comparative data sets are needed, particularly among patients with Child-Pugh C cirrhosis who may benefit from alternative surveillance strategies.

© 2025 The Author(s). Published by Elsevier B.V. on behalf of European Association for the Study of the Liver (EASL). This is an open access article under the CC BY license (http://creativecommons.org/licenses/by/4.0/).

Introduction

Hepatocellular carcinoma (HCC) is the fourth leading cause of cancer-related death globally,[1] and its incidence rate is projected to increase over the next decade.[2] If HCC is detected at an early stage, curative therapy is possible, with a 5-year survival of ∼70%. However, curative therapies are not available at later stages and median survival falls to <1 year.[3]

To improve early-stage HCC detection, professional society guidelines recommend semi-annual ultrasound (US)-based surveillance of patients with cirrhosis and subgroups with chronic HBV infection.[4,5] High sensitivity of surveillance modalities is crucial to detect small, potentially curable tumors, and high specificity is required to minimize false positive results and associated effects.[6–8] Although US has a specificity of 92%, its sensitivity alone for early-stage HCC detection is only 45%.[9] Adding α-fetoprotein (AFP) to US improves the sensitivity to 63%, but with a lower specificity of 84%.[9] Factors associated with suboptimal performance of US-based surveillance include truncal obesity, steatotic liver disease, and increased liver nodularity.[10–12] US performance is also significantly affected by operator expertise.[13] Accordingly, other surveillance tools, such as computed tomography (CT) or MRI, are increasingly considered. However, CT is associated with radiation exposure, and standard diagnostic liver MRI has drawbacks related to long procedural time and cost.[14,15] Nonetheless, MRI is increasingly used in the clinic given concerns about US performance.[16,17] In particular, abbreviated

* Corresponding author. Address: Division of Digestive and Liver Diseases, University of Texas Southwestern, 5959 Harry Hines Blvd, POB 1, Suite 420, Dallas, TX 75390-8887, USA. Tel.: +1 214 645 6029; fax: +1 214 645 6294.
E-mail address: amit.singal@utsouthwestern.edu (A.G. Singal).
† Co-first authors.
‡ Co-senior authors.
https://doi.org/10.1016/j.jhepr.2025.101357

MRI (AMRI), which has a shorter protocol (~15 min *vs.* >30 min for a full diagnostic liver MRI) that only includes the pulse sequences necessary for HCC detection, could be cost-effective for HCC surveillance.[18] The concept of AMRI has gradually gained acceptance as a potential surveillance tool. Meta-analyses suggest that AMRI has diagnostic potential independent of protocol variations, with pooled sensitivity and specificity estimates of 86% and 94-96%, respectively.[19] A study of prospectively recruited patients suggested that AMRI sensitivity was significantly higher than that of US (86.0% *vs.* 27.9%; p <0.001).[20] However, existing data are largely limited by single-center designs, spectrum bias given inclusion of all HCC stages, and lack of direct comparative data for AMRI *vs.* US in the same patient population. Therefore, our study directly compared the detection sensitivity and specificity of dynamic contrast-enhanced (DCE)-AMRI to that of US among patients with and without early-stage HCC.

Methods

Patient population

We conducted a retrospective case–control study at three US academic health centers from the North American Liver Cancer Consortium:[21,22] UT Southwestern Medical Center, Parkland Health, and University of Michigan. Each participating center has hepatology subspecialty clinics for patients with cirrhosis and a multidisciplinary liver tumor program for patients with HCC; two of the centers also have liver transplantation programs.[23] All three centers follow the American College of Radiology Liver Imaging Reporting and Data System (LI-RADS) guidelines for HCC imaging and diagnosis.[24,25]

We identified patients with cirrhosis who underwent a standard gray-scale liver US and a DCE liver MRI within a 6-month period between January 2012 and December 2019. We excluded patients in whom the imaging technique deviated from LI-RADS recommendations (*e.g.* non-contrast exam) or used hepatobiliary contrast for MRI, whose imaging study was unavailable for review, or who received HCC treatment before imaging. Cirrhosis was defined using the following criteria: histopathological confirmation, imaging findings indicative of cirrhosis (*i.e.* cirrhotic liver morphology with evidence of portal hypertension), or confirmatory non-invasive markers of cirrhosis, such as transient elastography, shear wave elastography, and blood-based biomarkers.

Cases were defined as those with early-stage HCC per Milan Criteria (single lesion ≥2 cm and ≤5 cm or up to three lesions, each ≥1 cm and ≤3 cm, with no evidence of macrovascular invasion or metastasis); HCC was defined by histopathology or LI-RADS diagnostic criteria (LR-5 lesion) on CT or MRI scans. CT or MRI was the imaging exam most proximate to HCC diagnosis, before any treatment, potentially including the MRI from which the AMRI was derived. Control patients had cirrhosis without HCC, confirmed through 12 months of clinical follow-up. This length of follow-up is sufficient to exclude HCC based on tumor doubling times.[26] The use of a case–control design follows the recommended strategy for validation of early cancer detection strategies,[27,28] which recommends a 'phase II' case–control study before validation in cohort studies. This study was approved by the institutional review board at each site.

US, AMRI, and radiological reporting

US exams were compliant with LI-RADS technical recommendations for screening liver US, which included gray-scale (B-mode) transverse and longitudinal views of each liver lobe.[25] Cinematic sweep of the liver was conducted as per routine practice. All MRI exams were compliant with LI-RADS technical recommendations[24] and included a large field-of-view coronal T2-weighted sequence (used for prescribing axial sequences), 2D axial T1-, T2-, and diffusion-weighted sequences, followed by DCE 3D axial T1-weighted sequence with fat suppression during the precontrast, arterial, portal, and delayed phases using gadobutrol at a weight-based dose of 0.01 mmol/kg (Gadavist, Bayer Healthcare, Whippany, NJ, USA). The dynamic AMRI protocol was simulated by extracting the coronal T2-weighted and DCE sequences; other sequences were excluded.

Two board-certified radiologists at each site (TY, MD, DF, and MM, each with 10 or more years of experience in abdominal MRI) were blinded to all clinical, laboratory, and imaging data, including the status of HCC diagnosis, and independently interpreted all US and AMRI examinations. Given that the same radiologists reviewed imaging at UT Southwestern and Parkland Health, these sites were combined for all analyses. To minimize reader bias, the order of image review was randomized between cases *vs.* controls as well as US *vs.* AMRI. For each modality, the radiologists recorded maximum diameter, location, and LI-RADS category of each liver observation using the LI-RADS v2017 US algorithm and the LI-RADS v2018 CT/MRI algorithm, respectively.

US examinations were categorized as positive if an US-3 observation was detected. AMRI examinations were categorized as positive if any liver observations with category LR-4, -5, or -M were identified, and negative otherwise. This threshold was chosen for AMRI because LR-1, 2, and 3 observations have low positive predictive values for HCC and, therefore, are likely to negatively impact specificity.[29–31] Furthermore, although patients with LR-3 lesions have an elevated incidence rate of HCC, it is fourfold lower than the rate observed in patients with LR-4 lesions.[32] Thus, we used the presence or absence of an LR-3 or LR-5/LR-M observation in sensitivity analyses.

Data collection

We manually abstracted demographic, clinical, and laboratory data for each patient from their electronic health records. Cirrhosis status was assessed by Child-Pugh class, and liver disease etiology was categorized as HCV, HBV, alcohol related, metabolic dysfunction-associated liver disease (MASLD), or other. Patients with Child-Pugh C cirrhosis were included given the interest in AMRI for HCC surveillance while awaiting liver transplantation. We recorded MRI indications, which were then categorized as follow-up of abnormal US, follow-up of abnormal AFP, follow-up of prior cross-sectional imaging, surveillance, and diagnostic.

Statistical analysis

We used logistic regression to identify variables associated with US and MRI detection performance, such as year of imaging exams and Child-Pugh score.[10,33,34] To mitigate

selection bias, we then matched cases and controls in a 1:2 ratio based on these variables using the cardinality matching algorithm, setting a caliper of 0.1 without replacement. This caliper was appropriate to ensure all subjects were matched in the setting of a significant association between matching and outcome variables.[35] After matching, we confirmed that all covariates achieved a standardized mean difference <0.1 (0.06 for Child-Pugh score and 0.08 for year of imaging exams).

Patient characteristics were summarized as medians with IQRs for continuous variables and with proportions for categorical variables. Using binary (positive *vs.* negative) categories for each modality, per-patient pooled reader sensitivity, specificity, positive predictive value, and negative predictive value were estimated, with their respective 95% CIs, using logistic regression with generalized estimating equations to account for correlations between multiple readers within a patient. We also reported the sensitivity and specificity of each individual reader with their respective 95% CIs. Inter-reader agreement between readers for US and AMRI positivity was computed using the Cohen κ statistic.[36] Cross-modality differences between US and AMRI sensitivities and specificities for early-stage HCC detection were assessed using patient-level bootstrap resampling of 10,000 replicates, and 95% CIs were calculated. We performed subgroup analyses to evaluate predictors of diagnostic performance, including patient characteristics (e.g., Child-Pugh class, BMI, and liver disease etiology) as well as MRI indication. We examined AMRI *vs.* US odds of patient-level sensitivity for early-stage HCC detection, overall and across patient subgroups. Differences were considered statistically significant if p <0.05. All statistical analyses were performed using R version 4.2.1 (R Foundation for Statistical Computing, Vienna, Austria).

Results

Patient characteristics

Between 2012 and 2019, 798 patients with cirrhosis had an US and a full MRI within 6 months of each other. After exclusion criteria (n = 29) and matching, 648 patients were included in the final analysis: 216 with early-stage HCC (cases) and 432 without HCC (controls) (Fig. S1).

Patient characteristics are presented in Table 1. Cases had a median age of 63 years (IQR 58–67), were majority male (75%), and were diverse regarding race and ethnicity (70% non-Hispanic White, 10% Hispanic, 15% Black, and 5% other) and liver disease etiology (62% viral hepatitis, 15% alcohol related, 14% MASLD, and 9% other). Controls had a median age of 59 years (IQR 53–65) and were also majority male (56%), and diverse regarding race and ethnicity (63% non-Hispanic White, 18% Hispanic, 12% Black, and 7% other) and liver disease etiology (56% viral hepatitis, 16% alcohol related, 15% MASLD, and 13% other). The proportions with Child-Pugh A class were 59% and 54% among cases and controls, respectively. Most cases (81%) had unifocal tumors, with a median maximum diameter of 2.4 cm (IQR 1.8–2.9). Most patients underwent MRI to follow up an US nodule; however, 44% of controls and 47% of cases underwent MRI for other reasons, most commonly for follow-up of abnormal AFP, follow-up of prior cross-sectional imaging (*e.g.* pancreatic cyst), or surveillance imaging. During the 12-month follow-up for controls, 182 (42%) had cross-sectional imaging (multi-phase CT scan or MRI), 206 (48%) had US surveillance, and 44 (10%) had no follow-up imaging. For cases, both MRI and US had a median time from imaging to HCC diagnosis within 1 month, with median times of 0 (IQR 0–0) and 28 (IQR 11–62) days, respectively. Among cases, HCC diagnosis was confirmed by histopathology in 32 patients (15%) and by imaging alone in 184 patients (85%).

US sensitivity for early-stage HCC detection

US patient-level sensitivity for HCC ranged from 67.3% to 79.4% between readers, with a pooled sensitivity of 71.1% (95% CI 66.6–75.2) (Table 2). US sensitivity estimates across patient subgroups are detailed in Table 3. Sensitivity was significantly lower in patients with Child-Pugh class B or C cirrhosis (59.8% *vs.* 78.6% for class A; p = 0.005). There was no significant difference in US sensitivity between patients who received MRI to follow up on an US nodule and those who underwent MRI for other reasons (74.8% *vs.* 66.8%, p = 0.30). US sensitivity was higher in cases confirmed by histopathology compared with those confirmed by imaging alone, although this difference was not statistically significant (81.3% *vs.* 69.3%, p = 0.16). Inter-reader agreement for US positivity was moderate to strong, with Cohen κ of 0.76 (95% CI 0.67-0.85) and 0.88 (95% CI 0.83-0.92) at sites 1–2 and 3, respectively.

AMRI sensitivity for early-stage HCC detection

Dynamic AMRI patient-level sensitivity ranged from 73.9% to 93.7% between readers using the LR-4 threshold, with a pooled sensitivity of 80.1% (95% CI 76.1–83.6). The pooled sensitivity of AMRI was significantly higher than that of US (p <0.001) (Fig. 1). Dynamic AMRI sensitivities, using the LR-4 threshold, across patient subgroups, are detailed in Table 4. Sensitivity was significantly lower in patients with Child-Pugh class C (52.6% *vs.* 84.7% for class A; p = 0.003). AMRI sensitivity did not differ significantly between patients who underwent MRI to follow up on an US nodule and those who received MRI for other reasons (84.8% *vs.* 74.8%, p = 0.09). AMRI sensitivity was significantly lower in cases confirmed by histopathology compared with those confirmed by imaging alone (62.5 *vs.* 83.2%, p = 0.005). Inter-reader agreement for dynamic AMRI positivity was moderate, with Cohen κ of 0.74 (95% CI 0.64–0.84) and 0.72 (95% CI 0.65–0.79) at sites 1–2 and 3, respectively. When using a stricter threshold of LR-5/M, AMRI pooled sensitivity was lower at 68.8% (95% CI 64.2–73.0), which did not significantly differ from US (p = 0.45). When using a lower threshold of LR-3, AMRI pooled sensitivity was higher at 93.5% (95% CI 88.3–96.5).

Comparison of US and dynamic AMRI sensitivity

Odds ratios (ORs) of AMRI *vs.* US patient-level sensitivity are depicted in Fig. 2. Across subgroups, the sensitivity of AMRI for early-stage HCC was consistently higher than that of US, except for patients with Child-Pugh class C (OR 0.51, 95% CI 0.14–1.92). In patients with Child-Pugh class A or B, the sensitivity of AMRI was significantly higher than that of US (OR 2.07, 95% CI 1.27–3.39). In the subgroup of patients whose US and AMRI were completed within 3 months of each other, AMRI continued to have higher sensitivity compared with US (OR 1.63, 95% CI 0.9–2.79). The sensitivity of AMRI was higher than

Table 1. Characteristics of patients with and without HCC.

Characteristic	Population	Patients without HCC (n_T = 432; n_{S1-2} = 138; n_{S3} = 294)	Patients with HCC (n_T = 216; n_{S1-2} = 63; n_{S3} = 153)	*p* value*	All patients (n_T = 648; n_{S1-2} = 201; n_{S3} = 447)
Age (years)†	T	59 (53–65)	63 (58–67)	<0.001	60 (55–66)
	S1–2	58 (50–65)	60 (57–66)		59 (53–65)
	S3	59 (54–65)	64 (60–69)		61 (56–66)
Sex				<0.001	
Male	T	242 (56)	161 (75)		403 (62)
	S1–2	76 (55)	52 (83)		128 (64)
	S3	166 (56)	109 (71)		275 (62)
Female	T	190 (44)	55 (25)		245 (38)
	S1–2	62 (45)	11 (17)		73 (36)
	S3	128 (44)	44 (29)		172 (38)
Race or ethnicity				0.04	
Black	T	52 (12)	32 (15)		84 (13)
	S1–2	22 (16)	20 (32)		42 (21)
	S3	30 (10)	12 (8)		42 (9)
Hispanic or Latino	T	76 (18)	22 (10)		98 (15)
	S1–2	66 (48)	19 (30)		85 (42)
	S3	10 (3)	3 (2)		13 (3)
Non-Hispanic White	T	272 (63)	151 (70)		423 (65)
	S1–2	38 (28)	22 (35)		60 (30)
	S3	234 (80)	129 (84)		363 (81)
Other‡	T	32 (7)	11 (5)		43 (7)
	S1–2	12 (8)	2 (3)		14 (7)
	S3	20 (7)	9 (6)		29 (6)
BMI†	T	28.5 (24.8–33.9)	27.8 (25.2–32.6)	0.10	28.3 (25.0–33.4)
	S1–2	28.4 (24.5–33.6)	26.8 (24.3–29.8)		27.8 (24.4–32.9)
	S3	28.7 (25.1–34.5)	28.6 (25.5–33.3)		28.6 (25.2–33.9)
Cause of liver disease				0.38	
Viral liver disease	T	243 (56)	135 (62)		378 (58)
	S1–2	67 (49)	44 (70)		111 (55)
	S3	176 (60)	91 (60)		267 (60)
Alcohol related	T	69 (16)	33 (15)		102 (16)
	S1–2	29 (21)	14 (22)		43 (21)
	S3	40 (14)	19 (12)		59 (13)
MASLD	T	66 (15)	29 (14)		95 (15)
	S1–2	25 (18)	4 (6)		29 (15)
	S3	41 (14)	25 (16)		66 (15)
Other	T	54 (13)	19 (9)		73 (11)
	S1–2	17 (12)	1 (2)		18 (9)
	S3	37 (12)	18 (12)		55 (12)
Child-Pugh class				0.18	
Child-Pugh A	T	225 (54)	124 (59)		349 (56)
	S1–2	66 (54)	37 (59)		103 (56)
	S3	159 (54)	87 (59)		246 (56)
Child-Pugh B	T	132 (32)	68 (32)		200 (32)
	S1–2	39 (32)	22 (35)		61 (33)
	S3	93 (32)	46 (31)		139 (31)
Child-Pugh C	T	59 (14)	19 (9)		78 (12)
	S1–2	17 (14)	4 (6)		21 (11)
	S3	42 (14)	15 (10)		57 (13)
AFP level (ng/ml)†	T	4 (2–7)	8 (4–28)	<0.001	5 (3–12)
	S1–2	4 (3–6)	12 (5–14)		5 (3–14)
	S3	4 (2–7)	7 (3–26)		4 (2–11)
Number of HCC lesions				–	
None	T	432 (100)	–		432 (67)
	S1–2	138 (100)	–		138 (69)
	S3	294 (100)	–		294 (66)
One	T	–	174 (81)		174 (27)
	S1–2	–	51 (81)		51 (25)
	S3	–	123 (80)		123 (28)
Two	T	–	33 (15)		33 (5)
	S1–2	–	10 (16)		10 (5)
	S3	–	23 (15)		23 (5)
Three	T	–	9 (4)		9 (1)
	S1–2	–	2 (3)		2 (1)
	S3	–	7 (5)		7 (1)

(*continued on next page*)

Table 1. (*continued*)

Characteristic	Population	Patients without HCC (n_T = 432; n_{S1-2} = 138; n_{S3} = 294)	Patients with HCC (n_T = 216; n_{S1-2} = 63; n_{S3} = 153)	*p* value*	All patients (n_T = 648; n_{S1-2} = 201; n_{S3} = 447)
Tumor diameter (cm)†	T	–	2.4 (1.8–2.9)	–	2.4 (1.8–2.9)
	S1–2	–	2.4 (1.9–3.1)		2.4 (1.9–3.1)
	S3	–	2.3 (1.8–2.9)		2.3 (1.8–2.9)
Year of imaging				0.67	
2012–2015	T	173 (40)	82 (38)		255 (39)
	S1–2	42 (30)	32 (51)		74 (37)
	S3	131 (45)	50 (33)		181 (40)
2016–2019	T	259 (60)	134 (62)		393 (61)
	S1–2	96 (70)	31 (49)		127 (63)
	S3	163 (55)	103 (67)		266 (60)

Unless otherwise specified, data are presented as n (%).
HCC, hepatocellular carcinoma; MASLD, metabolic dysfunction-associated steatotic liver disease; S1–2, sites 1–2; S3, site 3; T, total cohort.
**P* value assessing the difference between patients with and without HCC using Pearson's chi-square test for proportions and Mann-Whitney *U* test for distributions (p <0.05 indicates a statistically significant difference). *p* values relate to the total cohort (T) numbers within each section.
†Data are presented as median (IQR).
‡Other races included Asian, American Indian or Alaskan, and Native Hawaiian or other Pacific Islander.

Table 2. Patient-level sensitivity and specificity of AMRI and ultrasound for early-stage HCC detection in patients with cirrhosis.

Metric	Reader	Ultrasound (reference)	AMRI (LR-4/5/M)	AMRI (LR-5/M)
Sensitivity	Pooled	71.1 (66.6–75.2)	80.1 (76.1–83.6) (*p* <0.001)	68.8 (64.2–73.0) (*p* = 0.45)
	Reader 1	74.6 (62.5–83.8)	93.7 (84.3–97.6) (*p* = 0.008)	77.8 (65.9–86.4) (*p* = 0.80)
	Reader 2	79.4 (67.6–87.6)	87.3 (76.6–93.5) (*p* = 0.30)	69.8 (57.5–79.9) (*p* = 0.29)
	Reader 3	69.9 (62.2–76.7)	73.9 (66.3–80.2) (*p* = 0.47)	69.3 (61.5–76.1) (*p* = 1.00)
	Reader 4	67.3 (59.5–74.3)	77.8 (70.5–83.7) (*p* = 0.04)	64.1 (56.2–71.3) (*p* = 0.59)
Specificity	Pooled	72.3 (69.3–75.2)	91.9 (89.9–93.5) (*p* <0.001)	95.3 (93.6–96.5) (*p* <0.001)
	Reader 1	67.4 (59.1–74.7)	89.1 (82.7–93.3) (*p* <0.001)	94.9 (89.7–97.6) (*p* <0.001)
	Reader 2	68.1 (59.9–75.4)	90.6 (84.5–94.5) (*p* <0.001)	94.9 (89.7–97.6) (*p* <0.001)
	Reader 3	75.5 (70.3–80.1)	89.8 (85.8–92.8) (*p* <0.001)	93.2 (89.7–95.6) (*p* <0.001)
	Reader 4	73.5 (68.1–78.2)	95.9 (93.0–97.7) (*p* <0.001)	97.6 (95.1–98.9) (*p* <0.001)

Sensitivities and specificities are estimated using a generalized estimating equation logistic regression model; p <0.05 indicates a statistically significant difference from ultrasound assessed by 95% bootstrap CIs of the cross-modality differences using the McNemar test. AMRI, abbreviated magnetic resonance imaging based on dynamic contrast-enhanced imaging; HCC, hepatocellular carcinoma; LR, HCC probability categories of Liver Imaging Reporting and Data Systems (LI-RADS).

that of US for patients with T1 HCC (OR 1.53, 95% CI 0.68–3.45) and those with T2 HCC (OR 1.72, 95% CI 1.00–2.95). The sensitivity of AMRI was higher than that of US for cases confirmed by imaging alone (OR 2.19, 95% CI 1.33–3.60); however its sensitivity did not differ significantly and, in fact, was lower in cases confirmed by histology (OR 0.38, 95% CI 0.12–1.20).

Lesions missed by US and AMRI were smaller than detected lesions. The median diameter of missed lesions was 2.2 cm (IQR 1.6–2.9) for US and 2.0 cm (IQR 1.5–2.5) for AMRI, while the median diameter of detected lesions was 2.5 cm (IQR, 1.9–3.0) for AMRI and 2.4 cm (IQR 1.9–2.9) for US.

US specificity

The patient-level specificity of US ranged from 67.4% to 75.5% across readers, with a pooled specificity of 72.3% (95% CI 69.3–75.2) (Table 2). US specificities across patient subgroups are detailed in Table 3. Specificity was significantly higher in patients with Child-Pugh class B or C (77.8% *vs.* 67.3% for class A; *p* = 0.02). Specificity was significantly lower in patients who underwent MRI to follow up on an US nodule (58.9% *vs.* 89.7% for those who received MRI for other reasons, *p* <0.001).

Dynamic AMRI specificity

The patient-level specificity of dynamic AMRI by reader ranged from 89.1% to 95.9%, with a pooled specificity of 91.9% (95% CI 89.9–93.5). AMRI pooled specificity was significantly higher than that of US (*p* <0.001) (Fig. 1). AMRI specificity estimates across patient subgroups are detailed in Table 4. There was no difference in AMRI specificity between patients who underwent MRI to follow up on an US nodule and those who received MRI for other reasons (91.2% *vs.* 92.9%, *p* = 0.60). When assessed at a threshold of LR-5/M observations, the dynamic AMRI pooled specificity increased to 95.3% (95% CI 93.6–96.5). When applying a lower threshold of LR-3, AMRI specificity was 71.6% (95% CI 67.6–75.4), which was not significantly different from that of US (*p* = 0.77), but was significantly lower than the specificity of AMRI at an LR-4 threshold (*p* <0.001).

Positive and negative predictive values

The pooled positive predictive value for dynamic AMRI was 83.2% (95% CI 79.3–86.5), significantly higher than that for US (56.2%; 95% CI 52.0–60.3; *p* <0.001). The pooled negative predictive value for dynamic AMRI was 90.2% (95% CI 88.1–92.0), significantly higher than that for US (83.3%; 95% CI 80.5–85.8; *p* <0.001).

Discussion

In this multicenter paired comparison study, we found that dynamic AMRI had higher per-patient sensitivity and specificity for detection of early-stage HCC compared with US in patients with cirrhosis. Our sensitivity and specificity estimates are

Table 3. Patient-level sensitivity and specificity of ultrasound (US-3) for early-stage HCC detection across patient subgroups.

	Patients with early-stage HCC			Patients without HCC		
Characteristic	Sensitivity (95% CI)	No. of patients*	*p* value	Specificity (95% CI)	No. of patients*	*p* value
Age						
≤60 years	65.8 (57.7–73.0)	48/73	Ref.	74.8 (70.1–78.4)	187/250	Ref.
>60 years	73.8 (68.4–78.6)	105.5/143	0.28	69.0 (64.0–73.5)	125.5/182	0.22
Sex						
Male	71.9 (66.7–76.5)	115/160	Ref.	70.6 (66.2–74.4)	171.5/243	Ref.
Female	70.0 (60.8–77.8)	38.5/55	0.93	74.7 (70.1–78.9)	142/190	0.39
BMI (kg/m^2)						
<25	70.8 (61.0–79.0)	34/48	Ref.	69.9 (63.5–75.7)	75.5/108	Ref.
≥25 and ≤30	73.5 (66.3–79.7)	61/83	0.90	67.9 (62.2–73.1)	95/140	0.84
>30	68.8 (61.5–75.3)	58.5/85	0.96	77.2 (72.6–81.2)	142/184	0.22
Child-Pugh class						
A	78.6 (73.1–83.3)	97.5/124	Ref.	67.3 (62.9–71.5)	151.5/225	Ref.
B	57.4 (48.9–65.4)	39/68	0.003	75.4 (69.8–80.2)	99.5/132	0.14
C	68.4 (52.2–81.1)	13/19	0.49	83.1 (75.2–88.8)	49/59	0.028
B or C	59.8 (52.3–66.8)	52/87	0.005	77.8 (73.3–81.7)	148.5/191	0.024
A or B	71.1 (66.4–75.4)	136.5/192	0.17	70.3 (66.9–73.6)	251/357	0.51
Liver disease etiology						
Viral liver disease	76.7 (71.3–81.3)	103.5/135	Ref.	72.4 (68.3–76.2)	176/243	Ref.
Alcohol related	65.2 (53.0–75.6)	21.5/33	0.26	74.6 (66.7–81.2)	51.5/69	0.83
MASLD	58.6 (45.7–70.5)	17/29	0.08	68.9 (60.6–76.2)	45.5/66	0.69
Other	60.5 (44.5–74.6)	11.5/19	0.22	73.2 (64.0–80.7)	39.5/54	1.00
Order of study done						
US before AMRI	71.1 (66.4–75.3)	141.5/199	Ref.	69.2 (65.6–72.5)	236.5/342	Ref.
AMRI before US	69.2 (49.5–83.8)	9/13	1.00	84.7 (78.5–89.4)	72/85	0.006
Time between US and AMRI						
<3 months	75.6 (70.7–79.9)	125.5/166	Ref.	68.8 (64.9–72.3)	203.5/296	Ref.
3–6 months	56.0 (46.2–6.4)	28/50	0.01	80.2 (75.0–84.5)	109/136	0.02
Tumor stage						
T1	58.8 (49.1–67.9)	30/51	Ref.	–	–	–
T2	74.9 (69.9–79.2)	123.5/165	0.04	–	–	–
MRI indication						
Follow-up US	74.8 (68.8–80.0)	86/115	Ref.	58.9 (54.4–63.1)	142/241	Ref.
Other	66.8 (60.1–73.0)	67.5/101	0.30	89.7 (86.2–92.4)	171/191	<0.001

Sensitivities and specificities are estimated using a generalized estimating equation logistic regression model. Results represent univariable, unadjusted analyses; *p* <0.05 indicates a statistically significant difference from the reference variable using the two-sample Z-test for proportions.
AMRI, abbreviated magnetic resonance imaging based on dynamic contrast-enhanced imaging; HCC, hepatocellular carcinoma; MASLD, metabolic dysfunction-associated steatotic liver disease; T1, single lesion <2 cm; T2, single lesion 2–5 cm or multiple HCC lesions; US, ultrasound.
*Number of patients correctly interpreted to total number of patients, averaged over two readers per site.

consistent with those reported in a meta-analysis (86% sensitivity; 94% specificity)[19] and further support the potential advantage of AMRI over US in HCC surveillance.[20]

We explored factors that could modulate the detection performance of US and AMRI. While liver disease etiology did not adversely affect AMRI performance, MASLD was associated with worse US performance, suggesting AMRI as a preferred alternative in this subgroup of patients, although subgroup results should be interpreted carefully in light of small sample sizes. Both US and AMRI performed well in patients with compensated cirrhosis, with no significant difference in test sensitivity. Although this may be related to the small sample size in this subgroup, US also performed the best in this subgroup; thus, the relative benefit of AMRI may be smaller. These data are important when considering cost-effectiveness and identifying target populations for AMRI *vs.* US. Conversely, decompensated cirrhosis was associated with worse performance for both modalities, highlighting a need for alternative surveillance strategies in this group.[12,34] There is growing interest in biomarker panels, such as Gender, Age, AFP-L3, AFP (GALAD), as well as 'liquid' biopsies using circulating tumor DNA, which appear to have consistent performance independent of liver disease severity.[37]

Each imaging modality has benefits and drawbacks, complicating use of a single optimal imaging method for surveillance in all patients.[38] US may be more accessible than AMRI, despite lower per-patient sensitivity. AMRI may be of benefit for patients in whom US is likely to be limited, such as those with MASLD or obesity.[39] For this reason, risk-stratified or precision surveillance strategies may be considered, such as AMRI-based surveillance for high-risk patients or those who are not ideal candidates for US-based surveillance.[40] The concept of selecting among various surveillance strategies is not new; MRI for breast cancer screening is recommended over mammography for women with dense breasts and those at high lifetime risk.[41] The value of selected surveillance strategies should be evaluated in the context of lower annual HCC incidence rates in MASLD than in viral hepatitis.[1] Ongoing clinical trials, including FASTRAK, MAGNUS-HCC, and MIRACLE-HCC, are evaluating the role of AMRI *vs.* US for HCC surveillance.

Surveillance effectiveness is also driven by low adherence in practice.[42,43] Patients report a preference for MRI-based surveillance over US, which may augment surveillance adherence.[44] Although not recommended by clinical practice guidelines, studies have shown increasing use of MRI over

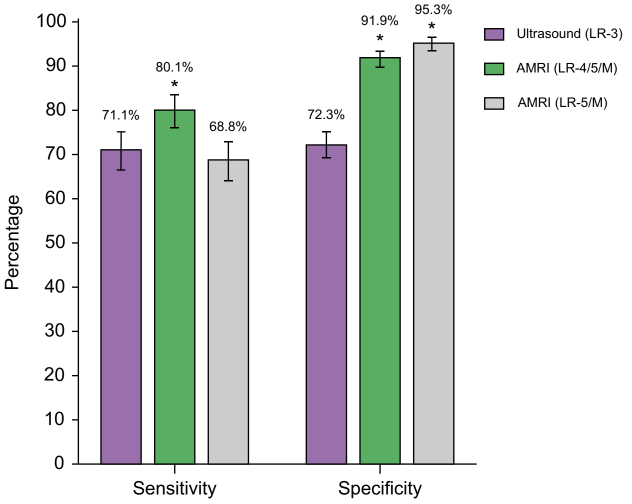

	Sensitivity		Specificity	
	No. of patients§	*p* value	No. of patients§	*p* value
Ultrasound (LR-3)	153.5/216	Ref.	312.5/432	Ref.
AMRI (LR-4/5/M)	173/216	<0.001	397/432	<0.001
AMRI (LR-5/M)	148.5/216	0.45	411.5/432	<0.001

Fig. 1. Patient-level sensitivity and specificity of AMRI and US for early-stage HCC detection in patients with cirrhosis. Sensitivities and specificities are estimated using a generalized estimating equation logistic regression model. *p <0.05 from US assessed by 95% bootstrap CIs of the cross-modality differences using the McNemar test. †Number of correctly interpreted patients to the total number of patients, averaged over two readers per site. AMRI, abbreviated magnetic resonance imaging; HCC, hepatocellular carcinoma; OR, odds ratio; US, ultrasound.

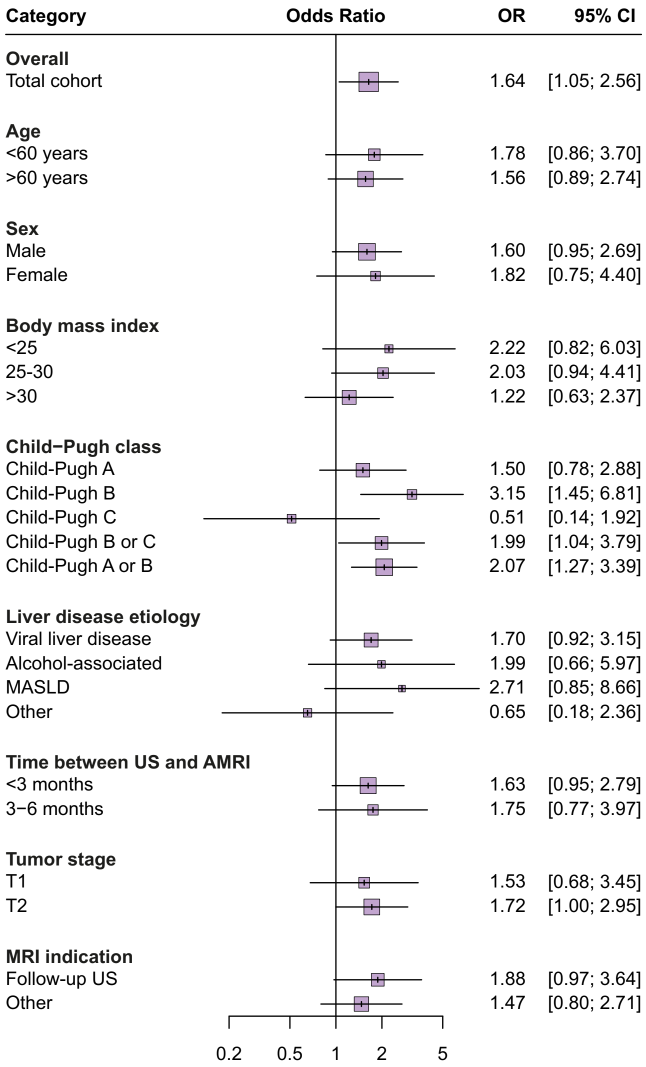

Fig. 2. AMRI *vs.* US odds of patient-level sensitivity for early-stage HCC detection, overall and across patient subgroups. ORs were calculated using the Mantel-Haenszel method for each subgroup. Results represent unadjusted comparisons across subgroups. ORs with 95% CIs that do not include 1 are statistically significant. AMRI, abbreviated magnetic resonance imaging; HCC, hepatocellular carcinoma; OR, odds ratio; US, ultrasound.

time.[16] Conversely, surveillance receipt may decrease in areas with limited MRI capacity or among low socioeconomic status populations, in whom financial burden can be a barrier to surveillance.[45,46] Future adoption of AMRI into guidelines and practice must consider several factors, including geographical differences in MRI capacity and access, financial barriers, contraindications to MRI (e.g., metal implants or claustrophobia), and need for further validation studies.[47,48]

In our study, we evaluated DCE-AMRI, although there are other AMRI protocols that have been proposed (e.g., non-contrast exams or AMRI with hepatobiliary agents). Results from a meta-analysis showed that there was no significant difference in the diagnostic performance of non-contrast-AMRI (sensitivity 86%; specificity 94%) and contrast-enhanced protocols (sensitivity 87%; specificity 94%) for HCC surveillance.[19] Although there is limited evidence to suggest an AMRI protocol over another based on diagnostic performance, it is important to consider the feasibility, adoption, availability, and cost of each strategy. For instance, although contrast-enhanced protocols appear to provide potential higher sensitivity over non-contrast-AMRI, the latter has a reasonable detection performance and provides notable benefits, such as eliminating the cost, risks, and practical considerations associated with contrast injection.[47,49] Gadolinium-based contrast agents can accumulate in the brain and other organs; although of unclear clinical significance, this is a potential consideration in patients undergoing repeated exposures for HCC surveillance.[48] However, DCE-AMRI provides the benefit of HCC detection and diagnosis during the same examination, which is not possible with other detection-only protocols, which would require repeat diagnostic MRI if positive.[47] Importantly, all AMRI protocols provide significant advantages over full MRI, including shorter acquisition time and shorter radiologist interpretation and reporting time, which may improve focus and efficiency.[38] Hence, the process of selecting the appropriate AMRI protocol will likely be multifactorial or institution-dependent.

We acknowledge various limitations of our study. First, this is a retrospective case–control study in patients who had contemporaneous US and MRI for clinical indications, most of whom (>80%) underwent MRI after US. This selection criterion was necessary to allow head-to-head cross-modality comparison, but it may have introduced a selection bias. Second, AMRI exams were simulated from full MRIs, and may not

Table 4. Patient-level sensitivity and specificity of AMRI (LR-4/5/M) for early-stage HCC detection across patient subgroups.

	Patients with early-stage HCC			Patients without HCC		
Characteristic	Sensitivity (95% CI)	No. of patients*	*p* value	Specificity (95% CI)	No. of patients*	*p* value
Age						
≤60 years	77.4 (69.9–83.5)	56.5/73	Ref.	91.2 (88.4–93.4)	228/250	Ref.
>60 years	81.5 (76.5–85.6)	116.5/143	0.60	92.9 (89.7–95.1)	169/182	0.66
Sex						
Male	80.3 (75.6–84.3)	128.5/160	Ref.	92.0 (89.2–94.1)	223.5/243	Ref.
Female	80.9 (72.5–87.2)	44.5/55	1.00	91.8 (88.6–94.2)	174.5/190	1.00
BMI (kg/m^2)						
<25	84.4 (75.7–90.4)	40.5/48	Ref.	92.1 (87.7–95.1)	99.5/108	Ref.
≥25 and ≤30	84.9 (78.7–89.6)	70.5/83	1.00	91.1 (87.1–93.9)	127.5/140	0.95
>30	72.9 (65.8–79.1)	62/85	0.19	92.4 (89.2–94.7)	170/184	1.00
Child-Pugh class						
A	84.7 (79.6–88.7)	105/124	Ref.	91.8 (88.9–94.0)	206.5/225	Ref.
B	80.8 (74.2–87.3)	55/68	0.64	93.9 (89.9–96.0)	124/132	0.59
C	52.6 (37.0–67.8)	10/19	0.003	90.7 (84.0–94.8)	53.5/59	0.99
A or B	83.6 (79.6–87.0)	160.5/192	0.92	92.4 (90.3–94.2)	330/357	0.90
B or C	74.7 (68.3–81.1)	65/87	0.10	92.9 (89.6–94.9)	177.5/191	0.80
Liver disease etiology						
Viral liver disease	84.8 (80.0–88.6)	114.5/135	Ref.	92.0 (89.2–94.0)	223.5/243	Ref.
Alcohol related	78.8 (67.3–87.0)	26/33	0.56	93.5 (88.0–96.6)	64.5/69	0.87
MASLD	79.3 (67.0–87.9)	23/29	0.65	92.4 (86.5–95.9)	61/66	1.00
Other	50.0 (34.6–65.4)	9.5/19	0.001	88.9 (81.5–93.6)	48/54	0.64
Order of study done						
US before AMRI	80.4 (76.2–84.0)	160/199	Ref.	92.7 (90.7–94.5)	317/342	Ref.
AMRI before US	76.9 (57.2–89.3)	10/13	1.00	88.2 (82.5–92.3)	75/85	0.26
Time between US and MRI						
<3 months	83.4 (79.0–87.1)	138.5/166	Ref.	92.4 (90.0–94.3)	273.5/296	Ref.
3–6 months	69.0 (59.3–77.3)	34.5/50	0.04	90.8 (86.8–93.7)	123.5/136	0.71
Tumor stage						
T1	68.6 (59.0–76.9)	35/51	Ref.	—	—	—
T2	83.6 (79.3–87.3)	138/165	0.03	—	—	—
MRI indication						
Follow-up US	84.8 (79.5–88.9)	97.5/115	Ref.	91.2 (88.3–93.4)	219.5/241	Ref.
Other	74.8 (68.3–80.3)	75.5/101	0.09	92.9 (89.8–95.1)	177.5/191	0.60

Sensitivities and specificities are estimated using a generalized estimating equation logistic regression model. Results represent univariable, unadjusted analyses; p <0.05 indicates a statistically significant difference from the reference variable using the two-sample Z-test for proportions.
AMRI, abbreviated magnetic resonance imaging based on dynamic contrast-enhanced imaging; HCC, hepatocellular carcinoma; MASLD, metabolic dysfunction-associated steatotic liver disease; T1, single lesion <2 cm; T2, single lesion 2–5 cm or multiple HCC lesions; US, ultrasound.
*Number of patients correctly interpreted to the total number of patients, averaged over two readers per site.

accurately represent the performance of prospectively performed AMRI exams. AMRI test performance may have been overestimated given the lack of independence from the gold standard diagnostic exam, although our estimates parallel results from a study using surgical pathology as the reference standard.[34] AMRI had numerically lower sensitivity compared with US in cases confirmed by histology; however, numbers were small and a previous study using pathology as a gold standard demonstrated higher sensitivity for AMRI across most examined subgroups. Third, because the study included two liver transplant centers, a higher proportion (>10%) with Child-Pugh class C cirrhosis was included compared with typical HCC surveillance programs. This may have resulted in underestimation of the sensitivity for both US and AMRI, because both modalities are limited in decompensated cirrhosis. However, these patients are an important population to understand, given the importance of early-stage HCC detection while awaiting liver transplantation. Fourth, our findings represent performance from a case–control study, which may overestimate absolute sensitivity and specificity estimates compared with a phase III biomarker cohort analysis; however, this should not significantly impact relative sensitivity and specificity differences between the modalities. Cohort studies will take years to complete, with a small number of incident early-stage HCCs. Thus, our study provides important data to guide clinical practice in the interim. Fifth, our subgroup results are limited by small sample sizes, which should warrant careful interpretation. Lastly, the AMRI and US exams were performed at large referral centers in the USA. US exams were performed by numerous sonographers, and images were interpreted by subspecialized radiologists serving on the HCC tumor boards at their respective institutions. Thus, because US is operator dependent, our performance estimates may not generalize to centers with lower patient volumes and less experience. Indeed, the sensitivity of US in our study was notably higher than that reported in the literature (71.1% *vs.* 45%, respectively).[9] Similarly, given that AMRI is also reader dependent, discrepancies in sensitivity may exist between our findings and those reported in the literature (80.1% *vs.* 86%, respectively).[19] Despite these limitations, our study provides one of the largest comparative analyses to date between US and AMRI performed within a short time frame within the same population of patients.

In conclusion, dynamic AMRI has significantly better sensitivity and specificity compared with US for the detection of early-stage HCC overall and across most patient subgroups, although its sensitivity may be lower in those with Child-Pugh class C cirrhosis. Future large prospective trials, as well as cost-effectiveness analyses, are needed to further clarify the optimal performance of AMRI, identify causes of underperformance, determine at-risk patients who may benefit the most from this approach, and characterize the feasibility and applicability of different abbreviation protocols.

Affiliations

[1]*Department of Internal Medicine, UT Southwestern Medical Center, Dallas, TX, USA;* [2]*Department of Radiology, UT Southwestern Medical Center, Dallas, TX, USA;* [3]*Department of Radiology, University of Michigan, Ann Arbor, MI, USA;* [4]*Department of Urology, University of Michigan, Ann Arbor, MI, USA;* [5]*Division of Gastroenterology, Kaiser Permanente Medical Group, Riverside, CA, USA;* [6]*Department of Internal Medicine, University of Michigan, Ann Arbor, MI, USA*

Abbreviations

AFP, α-fetoprotein; AMRI, abbreviated magnetic resonance imaging; CT, computed tomography; DCE, dynamic contrast-enhanced; GALAD, Gender, Age, AFP-L3, AFP; HCC, hepatocellular carcinoma; LI-RADS, Liver Imaging Reporting and Data System; MASLD, metabolic dysfunction-associated steatotic liver disease; MRI, magnetic resonance imaging; OR, odds ratio; US; ultrasound.

Financial support

AGS is supported by National Cancer Institute grants U01 CA271887, U01 CA230694, and R01 CA222900. NER is supported by National Cancer Institute grant K08CA259536. Research by MSD is conducted with support from CPRIT RP210041. The content is solely the responsibility of the authors and does not necessarily represent the official views of the National Institutes of Health. The funding agencies had no role in the design and conduct of the study; collection, management, analysis, and interpretation of the data; or preparation of the manuscript.

Conflicts of interest

AGS has served as a consultant or on advisory boards for Bayer, FujiFilm Medical Sciences, Exact Sciences, Universal Dx, Glycotest, Roche, Freenome, Delfi, GRAIL, Genentech, AstraZeneca, Eisai, Exelixis, Boston Scientific, HistoSonics. NDP has served as a consultant or on advisory boards for Bayer, FujiFilm Medical Sciences, Exact Sciences, Glycotest, and Freenome. MSD is a consultant for Covera Health, treasurer for the Society of Advanced Body Imaging, and receives royalties from Wolters Kluwer. NER has served as consultant for AstraZeneca. DTF has served on the advisor board for GE HealthCare and Philips Healthcare, and has active research agreements with GE HealthCare, Philips Healthcare, and Siemens Healthineers. TY has served on an advisory board of Guerbet and speaker panels for Siemens Healthineers and Northwest Imaging, as well as active research agreements with Siemens Healthineers. The remaining authors declare no conflicts of interest that pertain to this work.

Please refer to the accompanying ICMJE disclosure forms for further details.

Authors' contributions

Conceptualization: AGS (lead), NDP. Data curation: TY (lead), KSED (lead); DD, MSD, DTF, MM-L, NER, EY, NDP, AGS. Investigation: NDP, AGS. Methodology: NDP, AGS. Project administration: NDP, AGS. Resources: NDP, AGS. Formal analysis: TK, KSED. Supervision: NDP, AGS. Writing – original draft: TY (lead), KSED (lead), NP, AGS. Writing – review & editing: TY (lead), KSED (lead), NDP (lead), AGS (lead), DD, MSD, DF, MM-L, NER, EY. Funding acquisition: AGS.

Data availability statement

The data supporting the findings of this study are available within the article and its supplementary materials. Further data related to the study are available from the corresponding author, upon reasonable request.

Supplementary data

Supplementary data to this article can be found online at https://doi.org/10.1016/j.jhepr.2025.101357.

References

Author names in bold designate shared co-first authorship

[1] Singal AG, Kanwal F, Llovet JM. Global trends in hepatocellular carcinoma epidemiology: implications for screening, prevention and therapy. Nat Rev Clin Oncol 2023;20:864–884.

[2] Rahib L, Wehner MR, Matrisian LM, Nead KT. Estimated projection of US cancer incidence and death to 2040. JAMA Netw Open 2021;4:e214708.

[3] Reig M, Forner A, Rimola J, et al. BCLC strategy for prognosis prediction and treatment recommendation: the 2022 update. J Hepatol 2022;76:681–693.

[4] European Association for the Study of the Liver. EASL Clinical Practice Guidelines: management of hepatocellular carcinoma. J Hepatol 2018;69:182–236.

[5] Singal A, Llovet JM, Yarchoan M, et al. AASLD guidance on prevention, diagnosis and treatment of hepatocellular carcinoma. Hepatology 2023;78:1922–1965.

[6] Singal AG, Patibandla S, Obi J, et al. Benefits and harms of hepatocellular carcinoma surveillance in a prospective cohort of patients with cirrhosis. Clin Gastroenterol Hepatol 2021;19:1925–19232.e1.

[7] Atiq O, Tiro J, Yopp AC, et al. An assessment of benefits and harms of hepatocellular carcinoma surveillance in patients with cirrhosis. Hepatology 2017;65:1196–1205.

[8] Narasimman M, Hernaez R, Cerda V, et al. Hepatocellular carcinoma surveillance may be associated with potential psychological harms of in patients with cirrhosis. Hepatology 2023;79:107–117.

[9] **Tzartzeva K**, **Obi J**, Rich NE, et al. Surveillance imaging and alpha fetoprotein for early detection of hepatocellular carcinoma in patients with cirrhosis: a meta-analysis. Gastroenterology 2018;154:1706–17018.e1.

[10] Simmons O, Fetzer DT, Yokoo T, et al. Predictors of adequate ultrasound quality for hepatocellular carcinoma surveillance in patients with cirrhosis. Aliment Pharmacol Ther 2017;45:169–177.

[11] **Chong N**, **Schoenberger H**, Yekkaluri S, et al. Association between ultrasound quality and test performance for HCC surveillance in patients with cirrhosis: a retrospective cohort study. Aliment Pharmacol Ther 2022;55:683–690.

[12] **Schoenberger H**, **Chong N**, Fetzer DT, et al. Dynamic changes in ultrasound quality for hepatocellular carcinoma screening in patients with cirrhosis. Clin Gastroenterol Hepatol 2022;20:1561. 9.e4.

[13] Fetzer DT, Browning T, Xi Y, et al. Associations of ultrasound LI-RADS visualization score with examination, sonographer, and radiologist factors: retrospective assessment in over 10,000 examinations. Am J Roentgenol 2022;218:1010–1020.

[14] Yoon JH, Lee JM, Lee DH, et al. A comparison of biannual two-phase low-dose liver CT and US for HCC surveillance in a group at high risk of HCC development. Liver Cancer 2020;9:503–517.

[15] **Kim SY**, **An J**, Lim YS, et al. MRI with liver-specific contrast for surveillance of patients with cirrhosis at high risk of hepatocellular carcinoma. JAMA Oncol 2017;3:456–463.

[16] Daher D, Seif El Dahan K, Cano A, et al. Hepatocellular carcinoma surveillance patterns and outcomes in patients with cirrhosis. Clin Gastroenterol Hepatol 2023;22:295–304.

[17] Joshi K, Mendler M, Gish R, et al. Hepatocellular carcinoma surveillance: a national survey of current practices in the USA. Dig Dis Sci 2014;59:3073–3077.

[18] Goossens N, Singal AG, King LY, et al. Cost-effectiveness of risk score-stratified hepatocellular carcinoma screening in patients with cirrhosis. Clin Transl Gastroenterol 2017;8:e101.

[19] Gupta P, Soundararajan R, Patel A, et al. Abbreviated MRI for hepatocellular carcinoma screening: a systematic review and meta-analysis. J Hepatol 2021;75:108–119.

[20] Park HJ, Kim SY, Singal AG, et al. Abbreviated magnetic resonance imaging vs ultrasound for surveillance of hepatocellular carcinoma in high-risk patients. Liver Int 2022;42:2080–2092.

[21] Singal AG, Rich NE, Mehta N, et al. Direct-acting antiviral therapy for hepatitis C virus infection is associated with increased survival in patients with a

history of hepatocellular carcinoma. Gastroenterology 2019;157:1253. 63.e2.
[22] Singal AG, Ghaziani TT, Mehta N, et al. Recall patterns and risk of primary liver cancer for subcentimeter ultrasound liver observations: a multicenter study. Hepatol Commun 2023;7:e0073.
[23] Yopp AC, Mansour JC, Beg MS, et al. Establishment of a multidisciplinary hepatocellular carcinoma clinic is associated with improved clinical outcome. Ann Surg Oncol 2014;21:1287–1295.
[24] American College of Radiology. LI-RADS: quality assurance for CT and MR imaging for hepatocellular carcinoma (HCC). www.acr.org/Clinical-Resources/Clinical-Tools-and-Reference/Reporting-and-Data-Systems/LI-RADS. [Accessed 28 February 2025].
[25] Morgan TA, Maturen KE, Dahiya N, et al. US LI-RADS: ultrasound liver imaging reporting and data system for screening and surveillance of hepatocellular carcinoma. Abdom Radiol 2018;43:41–55.
[26] Nathani P, Gopal P, Rich N, et al. Hepatocellular carcinoma tumour volume doubling time: a systematic review and meta-analysis. Gut 2021;70:401–407.
[27] Singal AG, Hoshida Y, Pinato DJ, et al. International liver cancer association (ILCA) white paper on biomarker development for hepatocellular carcinoma. Gastroenterology 2021;160:2572–2584.
[28] Pepe MS, Etzioni R, Feng Z, et al. Phases of biomarker development for early detection of cancer. J Natl Cancer Inst 2001;93:1054–1061.
[29] **van der Pol CB**, **Lim CS**, Sirlin CB, et al. Accuracy of the Liver Imaging Reporting and Data System in computed tomography and magnetic resonance image analysis of hepatocellular carcinoma or overall malignancy-a systematic review. Gastroenterology 2019;156:976–986.
[30] Arvind A, Joshi S, Zaki T, et al. Risk of hepatocellular carcinoma in patients with indeterminate (LI-RADS 3) liver observations. Clin Gastroenterol Hepatol 2023;21:1091. 3.e3.
[31] Kanneganti M, Marrero JA, Parikh ND, et al. Clinical outcomes of patients with liver imaging reporting and data system 3 or liver imaging reporting and data system 4 observations in patients with cirrhosis: a systematic review. Liver Transpl 2022;28:1865–1875.
[32] Singal AG, Parikh ND, Shetty K, et al. Natural history of indeterminate liver nodules in patients with advanced liver disease: a multicenter retrospective cohort study. Am J Gastroenterol 2024;119:2251–2258.
[33] Colli A, Fraquelli M, Casazza G, et al. Accuracy of ultrasonography, spiral CT, magnetic resonance, and alpha-fetoprotein in diagnosing hepatocellular carcinoma: a systematic review. Am J Gastroenterol 2006;101:513–523.
[34] Yokoo T, Masaki N, Parikh ND, et al. Multicenter validation of abbreviated MRI for detecting early-stage hepatocellular carcinoma. Radiology 2023;307:e220917.
[35] Lunt M. Selecting an appropriate caliper can be essential for achieving good balance with propensity score matching. Am J Epidemiol 2014;179:226–235.
[36] Landis JR, Koch GG. The measurement of observer agreement for categorical data. Biometrics 1977;33:159–174.
[37] Parikh ND, Tayob N, Singal AG. Blood-based biomarkers for hepatocellular carcinoma screening: approaching the end of the ultrasound era? J Hepatol 2022;78:207–216.
[38] An JY, Pena MA, Cunha GM, et al. Abbreviated MRI for hepatocellular carcinoma screening and surveillance. Radiographics 2020;40:1916–1931.
[39] Park HJ, Seo N, Kim SY. Current landscape and future perspectives of abbreviated MRI for hepatocellular carcinoma surveillance. Korean J Radiol 2022;23:598–614.
[40] Kao S-YZ, Sangha K, Fujiwara N, et al. Cost-effectiveness of a precision hepatocellular carcinoma surveillance strategy in patients with cirrhosis. eClinicalMedicine 2024;75:102755.
[41] Saslow D, Boetes C, Burke W, et al. American Cancer Society guidelines for breast screening with MRI as an adjunct to mammography. CA Cancer J Clin 2007;57:75–89.
[42] Wolf E, Rich NE, Marrero JA, et al. Use of hepatocellular carcinoma surveillance in patients with cirrhosis: a systematic review and meta-analysis. Hepatology 2021;73:713–725.
[43] **Marquardt P**, **Liu P-H**, Immergluck J, et al. Hepatocellular carcinoma screening process failures in patients with cirrhosis. Hepatol Commun 2021;5:1481–1489.
[44] **Woolen SA**, **Singal AG**, Davenport MS, et al. Patient preferences for hepatocellular carcinoma surveillance parameters. Clin Gastroenterol Hepatol 2022;20:204. 15.e6.
[45] Singal AG, Tiro JA, Murphy CC, et al. Patient-reported barriers are associated with receipt of hepatocellular carcinoma surveillance in a multicenter cohort of patients with cirrhosis. Clin Gastroenterol Hepatol 2021;19:987–995.e1.
[46] Narasimman M, Hernaez R, Cerda V, et al. Financial burden of hepatocellular carcinoma screening in patients with cirrhosis. Clin Gastroenterol Hepatol 2023;22:760–767.
[47] Vietti Violi N, Taouli B. Abbreviated MRI for HCC surveillance: is it ready for clinical use? Eur Radiol 2020;30:4147–4149.
[48] Pavlides M, Francis S, Barnes E. Abbreviated MRI to screen for HCC in patients with cirrhosis. A step forward but a long road ahead. J Hepatol 2022;76:981–982.
[49] **Kim DH**, **Yoon JH**, Choi MH, et al. Comparison of non-contrast abbreviated MRI and ultrasound as surveillance modalities for HCC. J Hepatol 2024;81:461–470.

Keywords: Hepatocellular carcinoma; Cirrhosis; Surveillance; Abbreviated MRI; Ultrasound.
Received 6 June 2024; received in revised form 6 February 2025; accepted 10 February 2025; Available online 12 February 2025

Journal of Hepatology, Volume *7*

Supplemental information

Multicenter evaluation of abbreviated MRI and ultrasound for detecting early-stage hepatocellular carcinoma

Karim Seif El Dahan, Takeshi Yokoo, Darine Daher, Matthew S. Davenport, David T. Fetzer, Mishal Mendiratta-Lala, Nicole E. Rich, Edward Yang, Neehar D. Parikh, and Amit G. Singal

Multicenter evaluation of abbreviated MRI and ultrasound for detecting early-stage hepatocellular carcinoma

Karim Seif El Dahan, Takeshi Yokoo, Darine Daher, Matthew S. Davenport, David T. Fetzer, Mishal Mendiratta-Lala, Nicole E. Rich, Edward Yang, Neehar D. Parikh, Amit G. Singal

Table of contents

Fig. S1……………………………………………………………………………………2

Figure S1: Patient Eligibility Flow Diagram

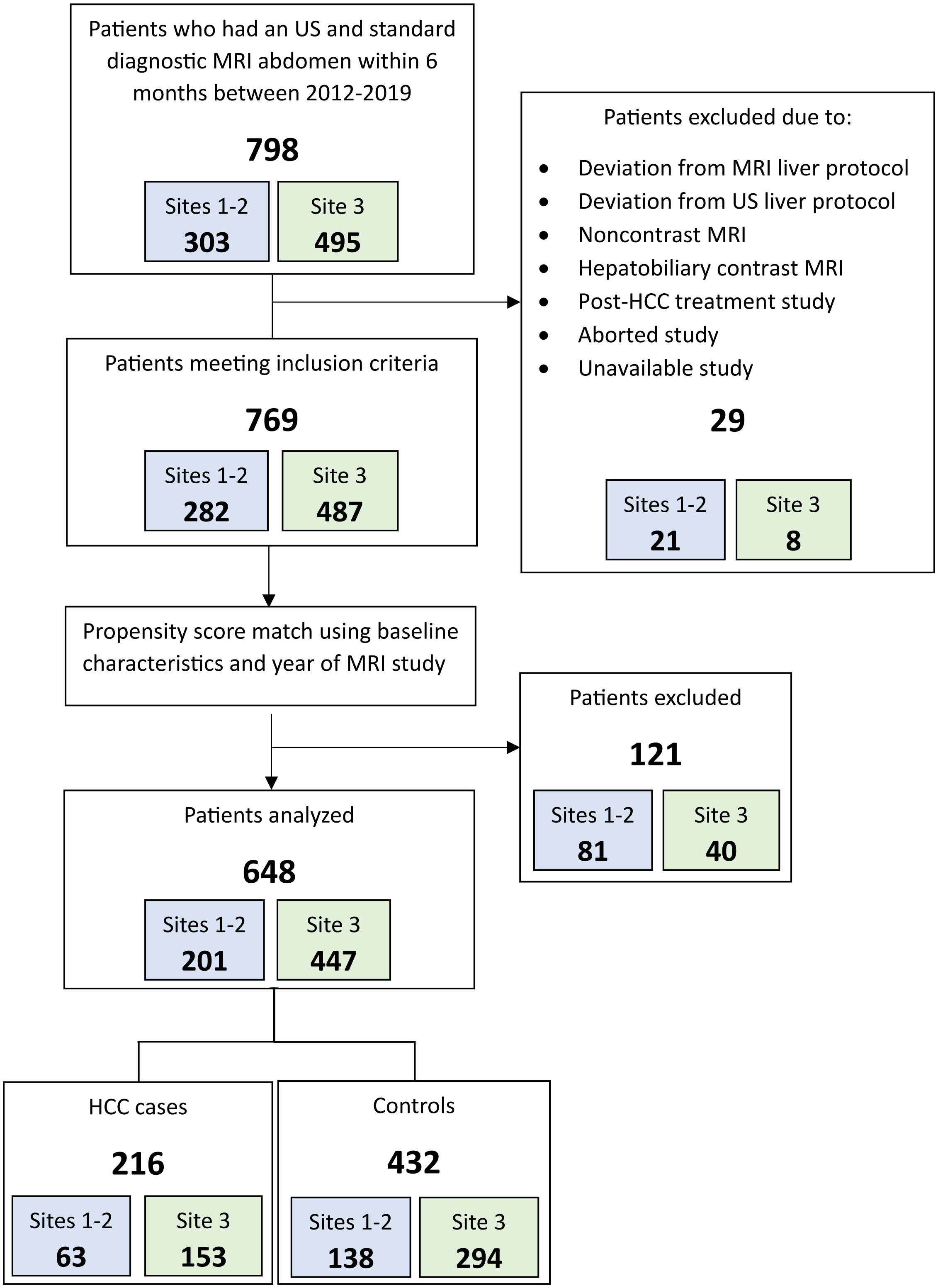

HCC = hepatocellular carcinoma.
